# Exploring the knowledge of community health workers about behavioral risk factors contributing to NCDs in Bushbuckridge, Ehlanzeni District, Mpumalanga Province, South Africa

**DOI:** 10.3389/fpubh.2026.1971276

**Published:** 2026-10-12

**Authors:** Thabo Difference Pilusa, Cairo Bruce Ntimana, Eric Maimela

**Affiliations:** 1 Department of Public Health, University of Limpopo, Sovenga, Limpopo, South Africa; 2 Dikgale Mamabolo Mothiba (DIMAMO) Population Health Research Centre, University of Limpopo, Polokwane, South Africa; 3 Department of Public Health, Walter Sisulu University, Butterworth, South Africa

**Keywords:** behavioral risk factors, chronic conditions, community health workers, knowledge, non-communicable diseases

## Abstract

**Background:**

Community health workers are essential in helping patients with non-communicable diseases; they must stay up to date on the latest information to carry out their duties efficiently. The present study aimed to explore CHWs' understanding of behavioral risk factors contributing to NCDs, providing valuable insights into their capacity to support NCD prevention and management, with a focus not only on behavioral risk factors but also on systemic and contextual challenges faced by CHWs.

**Methods:**

The study explores community health workers' knowledge about behavioral risk factors contributing to non-communicable diseases. Purposive sampling was used to select 20 CHWs. The study employed a qualitative, explorative, and descriptive design, and focus group discussions were used. Using thematic analysis, the researchers were able to organize and interpret the data presented in themes systematically.

**Results:**

Two themes emerged (Behavioral Risk Factors and Health System Analysis), along with seven subthemes, regarding the knowledge of community health workers about behavioral risk factors contributing to non-communicable diseases. The subthemes included unhealthy eating habits and poor lifestyle choices, mental health issues and stress, socio-economic influences, prevention support for lifestyle-related diseases, collaboration among healthcare professionals and stakeholders, conducting home visits, and logistical and structural challenges.

**Conclusion:**

The findings of the present study highlighted factors contributing to non-communicable diseases, including unhealthy diets, excessive alcohol consumption, and stress, particularly among women, as well as systemic barriers like financial constraints and long lines at the clinic that hinder treatment adherence. Community health workers emphasized the importance of prevention through health education on nutrition, stress management, and physical activity, along with collaborative efforts with healthcare professionals to manage chronic conditions effectively.

## 1 Introduction

Community health workers are the first responders, as they are the intermediaries between health/social services and the community to facilitate access to services and improve the quality and cultural competence of service delivery (1–3). In the global health workforce, CHWs are invaluable, acting as key facilitators of patient care, counseling, health education, healthy lifestyle promotion, and effective communication with the healthcare system (4–6). Many countries, particularly in developing regions, have integrated CHWs into their healthcare systems after recognizing their critical role in addressing community health needs (7–9).

Regardless of their essential function in addressing community issues, Community health workers encounter difficulties (10, 11). Several studies have emphasized that certain conditions, such as psychological disorders, are not yet considered non-communicable diseases (NCDs) but are rather correlated with the practice of witchcraft, bringing out a research gap in their understanding of behavioral risk factors for NCDs (5, 12–14). Previous studies have reported that CHWs are more than capable of delivering patient health outcomes when compared to trained healthcare workers (e.g., nurses) in areas like hypertension and diabetes control, with models developed for integrating CHWs into primary healthcare systems (15, 16). Despite their demonstrated efficacy, healthcare systems remain overburdened by the dual demands of infectious diseases and the growing prevalence of NCDs (17, 18). Insufficient infrastructure, limited human resources, and inadequate training further strain the system's capacity to provide appropriate counseling about NCDs and their associated risk factors (19, 20).

Given these challenges, employing a specialized team of healthcare professionals to support NCD management may be necessary. CHWs, with their accessibility and community-based approach, are uniquely positioned to assist patients in managing their illnesses effectively (21). While there is substantial literature on the role of CHWs in combating NCDs, there is a lack of literature in South Africa's Mpumalanga province regarding the knowledge, perspectives, and perceptions of CHWs on behavioral risk factors for NCDs. Against this background, the present study aimed to explore CHWs' understanding of behavioral risk factors contributing to NCDs, providing valuable insights into their capacity to support NCD prevention and management with a focus not only on behavioral risk factors but also on systemic and contextual challenges faced by CHWs.

## 2 Material and methods

### 2.1 Research design

This study employed a qualitative, exploratory, descriptive, and phenomenological design, which was suitable for capturing the subjective experiences, interpretations, and contextual knowledge of CHWs regarding behavioral risk factors and systemic barriers (22). The University of Limpopo Turfloop Campus approved the study protocol (Project number: TREC/1777/2023:PG), and the Mpumalanga Provincial Health Research and Ethics Committee (MPHREC) Reference Number: MP 202403 001 granted permission to conduct the study in a healthcare facility.

### 2.2 Study setting, study population, and sampling

The study was conducted in the Cottondale local area of the Bushbuckridge sub-district of Mpumalanga Province. The local area consists of six fixed clinics and one district hospital (District Health Information System, 2017). The Ehlanzeni District Municipality is situated in the northeast of the Mpumalanga Province. The target population of the study was community health workers selected from healthcare facilities in Bushbuckridge, Ehlanzeni District, Mpumalanga Province. Purposive sampling was utilized to select community health workers. Facility managers assisted in identifying eligible CHWs based on their experience in chronic disease support, and invitations were extended through in-person meetings and phone calls. CHWs with experience exceeding 2 years of assisting in the treatment and prevention of NDCs were included in the study.

### 2.3 Data collection

Data was collected through focus group discussions between May and August 2024. Three principal authors (TDP, CBN, and EM) conducted the focus group discussions with CHWs. Before the interviews were done, the primary authors clarified the reason for conducting the interviews, the voluntary nature of participation, and the freedom to withdraw at any moment. All the participants who took part in the study have signed an informed consent form. The interviews were conducted following the interview guide, with questions asked (see Supplement 1). Researchers recorded the sessions, which each lasted around 40 min, using audio recorders and notepads. Data collection continued until data saturation was reached on participant number 20.

### 2.4 Data analysis

The data analysis followed Tesch's open thematic approach, applied at multiple levels to text data provided by patients (23). Two authors (CBN and EM) organized the dataset, which comprised verbatim transcriptions of recorded audio files and accompanying field notes. The transcripts were anonymized prior to being uploaded into QSR NVivo 10 (QSR International, Warrington, UK) to facilitate the analysis process. To ensure a comprehensive understanding, the authors thoroughly reviewed the transcripts before proceeding to code the data. This involved identifying meaningful segments and assigning appropriate code labels. To ensure reliability, two researchers independently coded the transcripts. Intercoder reliability was established through comparison and discussion of code applications, with agreement reaching over 85%. Discrepancies were resolved through consensus, with input from a third author when necessary. The codes were then analyzed to identify and refine emerging themes. A collaborative discussion with the other authors helped to further refine and finalize these themes. The findings, presented in Table 1, represent the qualitative results, with all authors reaching consensus on the identified themes and subthemes. Tesch's open thematic approach provided a structured framework for coding and categorizing the data, allowing the authors to engage deeply with the material and uncover new insights. The final themes and subthemes are in Table 1.

**Table 1:** Summary of themes and sub-themes reflecting the findings from the interviews.

Themes	Sub-themes
1. Behavioral risk factors	1.1 Unhealthy eating habits and poor lifestyle choices. 1.2 Mental health problems and stress. 1.3 Socio-economic factors
2. Health system analysis	2.1 Assistance in the prevention of lifestyle diseases. 2.2 Collaboration between health care workers and other stakeholders. 2.3 Conducting home visits. 2.4 Logistics and structural challenges.

## 3 Results

The table presents a diverse group of women with varying ages, educational backgrounds, and marital statuses, highlighting the range of experiences and circumstances within this set of community health workers (Table 2).

**Table 2:** Characteristics of community health workers.

Pseudo names	Age	Gender	Study level	Marital status
CHW 1	47	Female	Grade 11	Unmarried
CHW 2	53	Male	Matric	Unmarried
CHW 3	40	Male	Matric	Married
CHW 4	53	Male	Grade 11	Married
CHW 5	49	Female	Grade 11	Married
CHW 6	53	Female	Matric	Married
CHW 7	53	Male	Matric	Married
CHW 8	53	Female	Grade 10	Widow
CHW 9	55	Female	Matric	Married
CHW 10	48	Female	Grade 11	Unmarried
CHW 11	50	Male	Matric	Unmarried
CHW 12	44	Female	Matric	Married
CHW 13	52	Male	Grade 11	Married
CHW 14	49	Male	Grade 11	Married
CHW 15	51	Female	Matric	Widow
CHW 16	45	Female	Grade 11	Married
CHW 17	47	Female	Matric	Married
CHW 18	54	Female	Grade 10	Widow
CHW 19	50	Female	Grade 11	Unmarried
CHW 20	46	Female	Matric	Unmarried

### 3.1 Theme 1: behavioral risk factors:

This theme focuses on exploring the various behavioral factors that contribute to Non-Communicable Diseases (NCDs) within the Bushbuckridge community. It involves studying lifestyle habits, such as diet, physical activity, smoking, and alcohol consumption, which have a significant impact on NCD prevalence. Understanding these factors can help in developing targeted interventions and preventive measures to address the root causes of NCDs within the Bushbuckridge community.

#### 3.1.1 Sub-theme 1.1: unhealthy eating habits and poor lifestyle choices

The participants discussed their individual experiences and insights into the potential causes of these illnesses. They described drinking too much Coca-Cola and eating foods that are rich in fat, sugar, and salt. One individual even attributed their stumbles and dizziness on drinking Coca-Cola. Furthermore, they mentioned that alcohol use is common in the community, even among children, which raises questions about how larger food and lifestyle choices may be causing health problems. This is what they had to say:

…*… “eating fat and salty diet” (CHW 6, aged 54)*

…*… “Tobacco smoking.” (CHW 5, aged 49)*

…*… “Alcohol consumption.” (CHW 9, aged 55)*

…*… “Thinking too much and not sharing what is troubling you to others, it makes to have mental pressure.” (CHW 2, aged 53)*

…*… “Raising hand, mmm, lack of exercise.” (CHW 3, aged 40)*

…*… “Another thing is failure to accept circumstances or situations.” (CHW 7, aged 53)*

…*… “People also don't eat enough fruits and vegetables.” (CHW 11, aged 50)*

…*… “There is a tendency to eat too much junk food because it is cheap.” (CHW 13, aged 52)*

…*… “Children as young as 10 years old are seen consuming alcohol regularly.” (CHW 15, aged 51)*

…*… “Smoking and drinking together make the body weak over time.” (CHW 18, aged 54)*

…*… “Snacking too much on chips and biscuits also adds to the problem.” (CHW 12, aged 44)*

#### 3.1.2 Sub-theme 1.2: mental health problems and stress

Participants shed light on the pervasive issue of mental health problems and stress, particularly among females. They highlighted a gendered aspect, suggesting that females tend to internalize stress more than males. Participants also expanded by indicating that stress often manifests as hypertension and is exacerbated by lifestyle factors.

…*… “Thinking too much and not sharing what is troubling you to others, it makes to have mental pressure.”(CHW 2, aged 53)*

…*… “Another thing is failure to accept circumstances or situations.” (CHW 7, aged 53)*

…*… “Women are overwhelmed by their family responsibilities.” (CHW 10, aged 50)*

…*… “Unemployment increases stress levels among men and women.” (CHW 14, aged 49)*

…*… “Some people turn to alcohol to cope with their problems, which worsens stress.” (CHW 19, aged 50)*

#### 3.1.3 Sub-theme 1.3: socio-economic factors

The participants indicated that participants usually face socioeconomic factors such as not being able to take medication on an empty stomach, living far from health care, and not having money for transport, and the difficulty of eating healthy food due to affordability. This is what they said:

…*… “Err, the challenge we face is that most of the patients say that they cannot take medication on an empty stomach.” (CHW 5, aged 49)*

…*… “Since the government have introduced a 6-month relief fund for the patient with chronic disease to help them to buy something to eat while taking medication, most of patient default treatment if they are better so that the relief fund can continue for more than 6 months.” (CHW 1, aged 47)*

…*… “It's hard to eat healthy food when you don't have money to buy it.” (CHW 8, aged 53)*

…*… “People live far from the clinics and struggle to afford transport.” (CHW 16, aged 45)*

### 3.2 Theme 2: health system analysis

#### 3.2.1 Sub-theme 2.1: assistance in prevention of lifestyle diseases

The participants in this study pointed out how they would emphasize the importance of health education, addressing diet, stress management, physical activity, and social support as important aspects. They illustrated the following:

…*… “Eish, we assist patients to be aware of danger of taking excessive fat diet and advice patients to reduce the intake of excessive salt diet.” (CHW 1, aged 47)*

…*… “Mm, to add on that, we encourage patients to avoid stress and also advise them to take a lot of water to reduce hypertension.”(CHW 2, aged 53)*

…*… “Yes, to add on that, we encourage patients to exercise and also if they have a problem or something bothering them, they need to share with others.” (CHW 3, aged 40)*

…*… “We explain how these habits can lead to long-term illnesses.” (CHW 17, aged 47)*

…*… “Some patients need constant reminders about exercise and eating habits.” (CHW 20, aged 46)*

#### 3.2.2 Sub-theme 2.2: collaboration between health workers and other stakeholders

Participants emphasized the importance of a team approach to managing chronic illnesses between community workers and healthcare professionals, with each group providing specialized roles and support to guarantee patients receive full treatment in the community.

…*… “Eish, we work together as a team, we refer patients to them, and they refer them back to us to assist them with taking of their medication at home. The health workers trained us how to give medication and how to identify signs and symptoms from the patient.” (CHW 1, aged 47)*

…*… “Mm, to add on that, they also supply us with materials such as clovers, machines and referral papers that we need to perform our work.” (CHW 5, aged 49)*

…*… “Err, we are working together for the benefit of the patient in case there is critically ill patient who needs a direct observation and or if the clinic is tracing the lost to follow-up patient, we are given the responsibility to trace the patient including their relatives.” (CHW 3, aged 40)*

…*… “To add on that, if we are in the community doing a door-to-door visits then when we find a patient seeking medical attention, we refer him/her to the clinic.” (CHW 2, aged 53)*

#### 3.2.3 Sub-theme 2.3: conducting home visits

Participants highlighted how they would facilitate assisting in access to healthcare services and ensure the wellbeing of patients by doing home visits.

…*… “We check on patients to ensure they are taking their medication.” (CHW 9, aged 55)*

…*… “During our visits, we also identify new cases needing medical attention.” (CHW 15, aged 51)*

#### 3.2.4 Sub-theme 2.4: logistics and structural challenges

Waiting in long queues can affect the decision to attend or not to attend health facilities. Moreover, it will mean that patients must pack up lunch boxes to sustain themselves throughout the day.

…*… “Most patients are complaining of long queues at the clinics.” (CHW 2, aged 53)*

…*… “The distance and time required discourage many from seeking care.” (CHW 18, aged 54)*

## 4 Discussions

The present study aimed to explore the knowledge of community health workers about behavioral risk factors contributing to NCDs. The CHW indicated that drinking too many soft drinks and eating foods that are rich in fat, sugar, and salt, and excessive alcohol consumption were associated with the development of NCDs. In agreement with the findings of the present study, several studies reported similar findings they indicating that sweet beverages were associated with a higher incidence of type 2 diabetes (24–26). Similarly, a study by Mokwena et al. (27), indicated that food high in fat and calories is associated with the risk of obesity and metabolic syndrome of which are risk factors for NCDs like diabetes and hypertension. These findings underscore a need to educate individuals about healthy dietary choices.

Participants highlighted the issue of mental health challenges and stress, emphasizing that females are more likely than males to internalize stress, which often manifests as hypertension and is aggravated by lifestyle factors. Participants highlighted the need for a gender-specific coping strategy. These findings are in agreement with Cheon et al. (28), They highlighted the need for tailored interventions and support networks for mental health and stress. These findings indicate that addressing mental health, in conjunction with physical health, is essential for effective chronic disease management.

CHWs highlighted significant challenges faced by patients with chronic diseases in adhering to treatment regimens, such as financial barriers to eating healthy and not being able to take medication on an empty stomach. These challenges underscore the importance of implementing integrated strategies to overcome them. In agreement with the findings of the present study, Grut et al. (29) highlighted that non-compliance with medication is not necessarily about neglect but could also be a matter of living in poverty. Similarly, a study by Neissen et al. (30), and Gatimu et al. (31), noted a correlation between lower income levels and the higher prevalence of hypertension in rural communities, suggesting that socioeconomic challenges exacerbate the burden of NCDs.

The findings of the present study also identified significant logistical and structural barriers within the healthcare system, particularly long queues at the clinics, and the distance and time required discourage many from seeking care. Participants advocated for the inclusion of home visits as a strategy to enhance healthcare access and promote early detection and intervention for chronic conditions. These findings are consistent with the work of Lizano-Diez et al. (32), who demonstrated the effectiveness of home-based chronic care management in improving patient outcomes. Additionally, the study by Breton et al. (33), highlighted system-level interventions, such as optimizing healthcare workflows and scheduling practices, as effective strategies for reducing waiting times and improving patient attendance. These findings underscore the urgent need to strengthen training and support systems for CHWs. Ongoing, structured training in NCD risk factors, including nutrition, stress, and mental health, will ensure that CHWs can provide accurate, evidence-based health education (34). In addition, providing psychosocial support, peer mentoring, and adequate resources (such as referral tools and transport allowances) will enhance CHWs' capacity to operate effectively in underserved communities (35). Recent global frameworks, including WHO's (36) guidance on optimizing CHW programs for NCD management, emphasize the need for standardized training, supervision, and integration into formal health systems. Aligning CHW practices in Bushbuckridge with these international recommendations could enhance service delivery and improve NCD outcomes in rural South Africa.

Despite the challenges faced by patients, i.e., mental health and stress, socio-economic factors, and significant logistical and structural barriers within the healthcare system, particularly long queues at the clinics and the distance and time required, discourage many from seeking care. Nonetheless, community health workers have implemented various strategies to prevent lifestyle diseases. They highlighted the importance of health education, focusing on diet, stress management, physical activity, and social support. They also collaborate with healthcare professionals to manage chronic illnesses and enhance patient access and wellbeing through home visits. These findings are in agreement with previous studies that reported community health workers (CHWs) being an integral part of the primary care worldwide (37–40). These findings suggest a critical need for regular training and capacity-building programs tailored to the realities of rural CHWs. Structured support systems, including refresher courses, mental health resources, and incentives, may improve CHWs' confidence and performance in managing NCDs (41, 42). Furthermore, health policy must prioritize integrating CHWs into decision-making processes to ensure low-level challenges are represented at the system level (43).

### 4.1 Study limitations

The viewpoints of the carefully chosen CHWs from the rural Black community who work in the healthcare systems in the Cottondale local area, which is in the Bushbuckridge sub-district of Mpumalanga Province, are represented in this study. It cannot, therefore, be widely applicable to other populations because it concentrates on a particular demographic and geographic location. Participants were selected from Bushbuckridge and other communities in the Ehlanzeni district municipality. In addition, given the predominance of female participants in this study, the findings may reflect gendered perceptions of health behaviors and stress responses. Female CHWs may be more attuned to issues affecting women, including internalized stress and caregiving burdens. Future studies should aim for a more gender-balanced sample to capture diverse perspectives. Although the study used rich qualitative data from CHWs, it did not incorporate additional sources such as field observations, patient records, or community health statistics that could have enabled triangulation. Incorporating such sources in future research would enhance the credibility and depth of the findings by validating CHWs' perceptions with valuable information. Nevertheless, the results provide information about what community health workers in rural South Africa know about behavioral risk factors that contribute to non-communicable diseases.

## 5 Conclusion

The findings of the present study highlighted factors contributing to non-communicable diseases, including unhealthy diets, excessive alcohol consumption, and stress, particularly among women, as well as systemic barriers like financial constraints and long lines at the clinic that hinder treatment adherence. Community health workers emphasized the importance of prevention through health education on nutrition, stress management, and physical activity, along with collaborative efforts with healthcare professionals to manage chronic conditions effectively. Based on the findings of the present study, the authors recommend strengthening the health education initiatives, improving access to affordable healthy foods and healthcare, addressing logistical challenges in service delivery, enhancing mental health support, and fostering collaboration among healthcare stakeholders for comprehensive care. While this study offers valuable insights from community health workers, it does not incorporate perspectives from patients or primary care providers. Including these stakeholders in future research would provide a more holistic understanding of how behavioral risk factors are managed and perceived across the healthcare facilities. Their insights would help validate CHWs' observations and strengthen the development of targeted interventions.

## Data Availability

The raw data supporting the conclusions of this article will be made available by the authors, without undue reservation.
