# Supplementary material for: Exploring the knowledge of community health workers about behavioral risk factors contributing to NCDs in Bushbuckridge, Ehlanzeni District, Mpumalanga Province, South Africa
Source: Front Public Health. 2026 Oct 12;14:1971276. doi: 10.3389/fpubh.2026.1971276 (PMC13696296; doi:10.3389/fpubh.2026.1971276)
Supplement: Supplementary file 1 [file Supplementary_File_1.docx]

**Appendix 1: Focus Group Discussion for Community Health Workers**

**HOME BASE CARERS/COMMUNITY HEALTH WORKERS/VOLUNTEERS**

**1. Role in the Community Health Workers** in relation to **prevention and control of non-communicable diseases.**

a. What do you think is your role in prevention, control of non-communicable diseases?
b. Do you think you are given the opportunity to play that role?
c. How do you relate with the health workers at the health facilities? **(Probe on referrals, supervision, supplies, reporting, etc).**

**2. Disease burden non-communicable disease in the area**

a. What are the most common non- communicable diseases in this area?
b. What are the main behavioural risk factors for the mentioned diseases? **(Probe on the causes for the various diseases)**
c. Which groups are the most affected?

**3. Community level concerns**

a. If given the opportunity, what questions would you like to ask Government officials and/or health care providers about health care delivery in this community in relation to prevention, control of non-communicable diseases? **(Probe for provision of health facilities, medicines, posting of health personnel, etc)**

**4. Community Health Care workers awareness of non-communicable disease prevention, control and management**

a. What is your view of the community’s use of your services in place?
b. What opportunities exist for CHWs in non-communicable disease prevention, control and management?
c. How are the CHWs in this community encouraged to take part in health service delivery?
d. How does CHWs get information on non-communicable disease prevention, control and management?

**(Probe on media, community meetings, etc).**

**5. Community Health workers participation in health service delivery in relation to non-communicable diseases prevention, control and management.**

a. How are CHWs involved in health service delivery?
b. How are CHWs organised to participate in health service delivery? Probe whether they are organised in committees, groups, etc
c. What is your view about the level of CHWs participation?

**(Probe on community-based insurance, voucher schemes, waivers).**

**6. Readiness (willingness and ability/capacity) of the communities to engage in health service delivery)**

a. How willing and ready are the CHWs in this community to get involved in health service delivery? **Please, give some examples.**
b. What training has been undertaken for CHWs to increase their participation in health activities?
c. What difficulties do people face in their participation in health care activities in this community?

**Appendix: Focus Group Discussion for Community Health Workers: Local language Sepedi.**

**1.Karolo yeo o ye bapalang** **mabapi le thibelo le taolo ya malwetši a go se alafšege.**

a. Re botše, o nagana gore o na le maikarabelo afe tabeng ya thibelo le taolo ya malwetši a go sa alafšege?
b. Na o nagana gore o fiwa sebaka sa go bapala karolo yeo?
c. Na o berekišana bjana le bašomi ba bangwe ba tša maphelo mo kliniking**? (Botšiša ka thomelo ya balweši, thlahlo, bjala bjalo)**

**2. Tshedimošo ya Bolwetši ga tikologo ya lena.**

a. Re botše gore ke malwetši afe a go sa alafšege oa a lego gona mo tikilogong ya lena?
b. Ke maitswharo afe ao o bonang eke a bala malwetši a go sa alafšege?
c. Ke bomang bao gantši ba swarago ke malwetši ao?

**3. Go kgomega ka setšhaba**

a. Ge o ka fiwa sebaka, ke potšišo efe yeo o dumago go botšiša bašomedi ba mmušo goba tša kalafo mabapi le thibelo le taolo ya malwetši a go se alafšege setšhabeng. **(Botšiša ka kabo ya dikliniki, dihlare le bašomi bjalo bjalo).**

**4. Tsebo goba maitemogelo ya lena mabapi le mekgwa yeo e bakago malwetši a go se alafšege.**

a. Re botše, go ya ka maitemogelo a gago, setšhaba se lebelela bjang mananeo a tša thibelo ao le se abelang o na

b. ke dibaka dife tšeo di leng gona tša lena tabeng ya thibelo le taolo ya malweši a go sa alafšege?

c. Lena le hlohleletšwa bjang gore se tšee karolo?

d. Lena le hwetša bjang tshedimošo ya mabapi le thibelo le taolo ya malweši a go sa alafšege? **( Botšiša ka tša kgokagano ya leago, dikopano tša setšhaba bjalo bjalo).**

**5. Kakaretsego ya community Health workers ga tša maphelo mabapi le mekgwa yeo e bakago malwetši a go se alafšege.**

a. lena le tšena bjang ga mananeo ga di tirelo tša maphelo?

b. Le rulagantšhwe bjang gore le be le karolo mananeo a tša kabo ya di tirelo? **(botšiša ge e ba a dirwa ka dihlopha, bjalo bjalo).**

c. Re botše, o bona nke o tšea karolo ga kakaa’ng ga mananeo a tša kalafo?

**6. Maikemišetšo a setšhaba ga tša maphelo mabapi le mekgwa yeo e bakago malwetši a go se alafšege.**

a. Na o ikemišeditše ga kakaa’ng gore o tšee karolo ditabeng tša ditirelo tša maphelo?

b. ke hlahlo efe yeo o e tsenetšego ya mabapi le thibelo ya malwetši a go sa alafšege?

c. ke dihlohlo dife tšeo le itemogetšego tšona tšeo batho ba lebeletšanago natšo tabeng ya tšea karolo ga mananeo a tša maphelo setšhabeng?
